# Nutritional Literacy Among Uninsured Patients With Diabetes Mellitus: A Free Clinic Study

**DOI:** 10.7759/cureus.16355

**Published:** 2021-07-13

**Authors:** Bianshly Rivera Rivero, Alena Makarova, Dina Sidig, Saniya Niazi, Rasha Abddelgader, Sabbir Mirza, Hadi Joud, Mustafa Urfi, Abdillahi Ahmed, Omar Jureyda, Firaas Khan, Justin Swanson, Maqsood Siddique, Natalia Weare-Regales, Abu-Sayeef Mirza

**Affiliations:** 1 Endocrinology, Diabetes and Metabolism, University of South Florida, Tampa, USA; 2 Medicine, University of South Florida Morsani College of Medicine, Tampa, USA; 3 General Medicine, Red Crescent Clinic, Tampa , USA; 4 General Medicine, Red Crescent Clinic, Tampa, USA; 5 Miscellaneous, University of South Florida College of Arts and Sciences, Tampa, USA; 6 Miscellaneous, University of South Florida College of Public Health, Tampa, USA; 7 Cardiology, James A. Hailey Veterans Affairs Medical Center, Tampa, USA; 8 Endocrinology, Diabetes and Metabolism, James A. Hailey Veterans Affairs Medical Center, Tampa, USA; 9 Internal Medicine, University of South Florida Morsani College of Medicine, Florida, USA

**Keywords:** diabetes mellitus, nutrition literacy, health literacy, nutrition, free clinics, uninsured

## Abstract

Objective

Evaluate nutrition literacy in uninsured subjects with diabetes mellitus (DM) who presented to free diabetes management classes.

Design

This single-site, cross-sectional observational study recruited thirty subjects from a free clinic for uninsured patients to attend diabetes mellitus, self-management classes. Before starting the classes, DM care-related data were collected, and subjects were administered the Nutrition Literacy Assessment Instrument (NLit). The assessment covers six subscales in nutrition and categorizes results into three possible categories: the likelihood of poor nutrition literacy (NLit Score ≤ 44), the possibility of poor nutrition literacy (NLit Score- 45-57), and the likelihood of good nutrition literacy (NLit score ≥ 58).

Results

Median glycated haemoglobin (HbA1c) was 7.45% for study participants. The mean NLit score was 38.1 (SD ± 9.4), correlating with a likelihood of poor nutrition literacy. All participants had either likelihood or the possibility of poor nutrition literacy based on the NLit Assessment. There were no participants who scored in the range of likelihood of good nutrition literacy. Subjects who scored in the range of likelihood of poor nutrition literacy had a significantly higher mean HbA1c (8.6 %) than those who scored in the possibility of poor nutrition literacy (6.9 %, p=0.005).

Conclusions

Poor nutrition literacy is associated with worse glycemic control among uninsured subjects with diabetes mellitus.

## Introduction

As of 2018, the Center for Disease Control and Prevention reported 34.2 million Americans with DM and 88 million with pre-diabetes. One out of ten Americans has diabetes, and one-third are at a high risk of developing diabetes. Furthermore, diabetes management issues have a direct impact on the health and socioeconomic wellbeing of our society. In 2017, the US's estimated total diabetes direct and indirect cost was $327 billion [1]. For individual patients, the cost burden of diabetes can be devastating. Patients with diabetes spend 2.3 times more on medical expenditures than non-diabetics [2]. Unfortunately, diabetes is also more prevalent in lower-income, minority groups, and the medically uninsured [3,4]. This latter group has been directly associated with poor diabetes outcomes [3,5]. Targeting behavioural factors and disease knowledge could lessen hyperglycemia, potentially reducing the morbidity, mortality, and cost associated with this disease among the uninsured, a poorly represented demographic in the medical literature.

Health literacy is a measure of a person's ability to obtain, read, understand, and use health information to make appropriate health decisions and to follow treatment recommendations [6]. Previous studies have shown that low health literacy is associated with poor glycemic control, poor diabetes knowledge, fewer self-management behaviours, and higher healthcare costs [7-11]. Furthermore, factors such as living in poverty, low education level, being part of a minority group, and lack of health insurance have all been linked to low health literacy [12]. The National Institutes of Health, Healthy People 2020, and the Institute of Medicine have identified poor health literacy to be a barrier to health. According to The US Office of Disease Prevention and Health Promotion, improvements in health practice that address low health literacy are needed to reduce disparities in health status [13].

An essential component of health literacy is nutrition literacy, defined as how individuals can obtain, process, and understand nutrition information [14,15]. It also measures the skills necessary to make appropriate nutritional decisions [16]. Our study measured nutrition literacy in a group of uninsured patients with diabetes who presented to a free educational diabetes self-management class. We hypothesized that uninsured patients with diabetes would have the likelihood or possibility of poor nutrition literacy, as previously published research reported low health literacy in patients who were uninsured and had lower socioeconomic status.

## Materials and methods

This single-site, cross-sectional observational pilot study describes the baseline characteristics, diabetes care-related data, and nutritional literacy of uninsured patients attending a monthly diabetes education class at a free clinic in Tampa, Florida, between October 2018 to March 2019. Uninsured men and women aged 18 years or older who had a diagnosis of DM were invited to participate in the study. Exclusion criteria included: insured subjects, pediatric subjects, and pregnant women. This free diabetes self-management class was publicly advertised as a University-funded resource that awarded voluntary participation with free supplies that included glucometers, test strips, and lancets. Subjects with diabetes, evaluated and managed at the free clinic, were encouraged and referred to attend this class. 

This study was approved by the University of South Florida Institutional Review Board with an expedited review (protocol #Pro00023920). Each patient was provided written informed consent to have recorded de-identified data collected via surveys utilizing portable electronic devices.

We gathered self-reported diabetes-care related data and the NLit Assessment answers. The NLit Assessment Instrument is a 64-item survey that has been validated and demonstrated to be reliable at measuring nutrition literacy (entire reliability 0.97; CI- 0.96-0.98 and test-retest reliability 0.88; CI, 0.85-0.90) [16]. The assessment covers six subscales: Nutrition & Health; Energy Sources in Food; Household Food Measurements; Food Label & Numeracy; Food Groups; and Consumer Skills. It categorizes results into three possible categories: the likelihood of poor nutrition literacy (NLit Score ≤ 44), the possibility of poor nutrition literacy (NLit Score 45-57), and the likelihood of good nutrition literacy (Nlit score ≥ 58) [16].

Data were collected and managed using REDCap electronic data capture tools hosted at the University of South Florida. REDCap (Research Electronic Data Capture) is a secure, web-based application designed to support data capture for research studies, providing our study with an intuitive interface for a validated data entry. All further data analyses were performed using SAS 9.4 (SAS Institute, Cary, NC). Differences in continuous outcomes between groups were tested using Welch's t-test for two groups and Welch's one-way analysis of variance (ANOVA) for three or more groups. A two-tailed value of p<0.05 was selected to indicate statistical significance [17].

## Results

Patient characteristics

This free clinic population has reported chronic disease incidence and socioeconomic factors in prior studies [4,18]. From October 2018 to March 2019, a total of 30 patients consented to participate in the survey before attending a free diabetes education class. The median age was 60 years old (n=30); 50% were female and 50% male (Table 1). Their education level ranged widely, from no education to post-graduate education. Most subjects had Type 2 DM (96%), and 50% were on oral medications. Subjects expressed that their barriers to diabetes care included the cost of medication (33%) and the cost of healthcare provider visits (33%). Only 10% of patients expressed that lack of education on diabetes treatment was a barrier (Table 1).

**Table 1:** Socioeconomic, anthropometric, and diabetes care-related data for adults participating in free diabetes self-management classes

Characteristic	Participants, n = 30, (%)
Age	
Median(range)	60 (29-70)
Sex	
Male sex	15 (50)
Female sex	15 (50)
Race	
Middle Eastern	13 (43)
Latino / Hispanic	2 (7)
Black	6 (20)
White	2 (7)
Asian-Indian Subcontinental	5 (17)
Other	2 (7)
Education level	
None	1 (3)
Elementary	1 (3)
Middle school	2 (7)
High school	8 (27)
College degree	12 (40)
Graduate school	4 (13)
Post-graduate	2 (7)
Employment	
Employed	13 (43)
Unemployed	17 (57)
Comorbidities	
Hypertension	19 (63)
Hyperlipidemia (treated with statin)	16 (53)
Exercise regimen	
Yes	22 (73)
No	8 (27)
Diabetes type	
Type 1	1 (3)
Type 2	29 (97)
Diabetes medication regimen	
No medication, lifestyle changes	8 (28)
Oral medications	15 (50)
Metformin	14 (47)
Sulfonylureas	4 (13)
DPP-4	1 (3)
GLP-1 agonist	0 (0)
Pioglitazone	0 (0)
SGLT2 inhibitor	1 (3)
Alpha-glucosidase inhibitor	6 (20)
Insulin only	4 (13)
Short-acting	4 (13)
Long-acting	4 (13)
Oral medications and insulin therapy	3 (10)
Other medications	6 (20)
Herbs / Supplements	7 (23)
Adherence to prescribed regimen	
Yes	18 (60)
No	12 (40)
Blood pressure medication	
Thiazide	5 (17)
Loop diuretic	0 (0)
Calcium channel blocker	7 (23)
ACE inhibitor	7 (23)
Angiotensin receptor blocker	3 (10)
Beta blocker	4 (13)
None	0 (0)
Statin use	
Yes	16 (53)
No	14 (47)
Aspirin use	
Yes	10 (33)
No	20 (67)
Barriers to diabetes care	
Local healthcare provider availability	5 (17)
Cost of healthcare provider visit	10 (33)
Transportation to healthcare provider	3 (10)
Availability of medications at pharmacy, hospital, health center, or mail	4 (13)
Cost of medications	10 (33)
Medication side effect concern	8 (27)
Lack of education on diabetes treatment	3 (10)
None	8 (27)
BMI	
Mean (SD)	26.6 (8.5)
Blood glucose	
Median (range)	140 (95 – 407)
HbA1C	
Median (range)	7.45 (6 – 11.7)

Nutrition Literacy

The mean score in the NLit was 38.1 (SD±9.4), which corresponds with a likelihood of poor nutrition literacy. The maximum score was 56, which corresponds with the possibility of poor nutrition literacy. There were no patients who scored in the range of likelihood of good nutrition literacy (Table 2). Gender, race, and education did not significantly impact NLit score.

**Table 2:** Mean Nutrition Literacy Assessment score based on sex, ethnicity, and education level for adults participating in free diabetes self-management classes

Characteristic	Participants (n = 30)
Mean NLit score (SD)	38.1 (9.4)
Sex	
NLit score	
Male sex	35.9
Female sex	40.3
p-value	0.204
Race	
NLit score	
Middle Eastern	39.4
Latino / Hispanic	45
Black	40.5
White	37.5
Asian-Indian Subcontinental	30.4
Other	35
p-value	0.397
Education level	
NLit score	
None	35
Elementary	37
Middle school	39
High school	39.3
College degree	38.4
Graduate school	36.5
Post-graduate	35.5
p-value	0.998

Patients scored the lowest in the domain of food numeracy and labels (Mean score: 4.2 SD ± 2.2) and scored the highest in food groups (mean score 11.7 SD± 3.6), compared to all other sections (Table 3).

**Table 3:** Nutrition Literacy Assessment results per domain for adults participating in free diabetes self-management classes

Domain Type	Participants (n = 30)
Nutrition and health	
Number of questions	10
Mean correct (SD)	6.2(2.2)
Energy Sources in Food	
Number of questions	10
Mean correct (SD)	5.9(2.3)
Household food measurement	
Number of questions	9
Mean correct (SD)	5.3(1.8)
Food label and numeracy	
Number of questions	10
Mean correct (SD)	4.2(2.2)
Food groups	
Number of questions	16
Mean correct (SD)	11.7(3.6)
Consumer skills	
Number of questions	9
Mean correct (SD)	4.7 (1.6)

Hemoglobin A1c negatively correlated to Nlit score (r= -0.24, CI -0.64 - 0.25, p=0.326), but this association did not reach statistical significance. However, patients who scored in the range of likelihood of poor nutrition literacy had a significantly higher mean HbA1c (8.6 %, mean difference 1.9, CI 0.6 - 3.0, p=0.005) than those who scored in the possibility of poor nutrition literacy (6.9%) (Table 4).

**Table 4:** Relationship between nutrition literacy and mean glycated haemoglobin for adults participating in free diabetes self-management classes

Nutrition Literacy	Participants (n = 30)
Possibility of poor literacy	
Mean A1C (SD)	6.7(0.3)
Likelihood of poor literacy	
Mean A1C (SD)	8.6(1.9)
Mean difference	1.9
95% CI of mean difference	(0.6-3.0)
p-value	0.005

## Discussion

There is a direct correlation between medical nutritional therapy and diabetes control [19]. The American Diabetes Association recommends that all patients with diabetes receive individualized medical nutrition therapy to promote healthy eating habits and improve HbA1c, blood pressure, and cholesterol levels. Nevertheless, it is essential to note there are intricate complexities to this task since dietary modification is dependent on many factors. These include health literacy, cultural preferences, personal preferences, access to healthy food choices, and willingness to change lifestyle [20]. Several scholars have proposed that nutrition literacy is a domain of health literacy [21]. 

Low health literacy has been described as a barrier to improving health outcomes in groups with ethnic or racial disparities [7]. Although low education has been associated with low health literacy [22,23], there was no significant difference in nutrition literacy based on education level or ethnicity in our cohort. Interestingly, none of the subjects scored in the highest category, the likelihood of good nutrition literacy. Most of them (n=22, 73%) scored in the lowest category, the likelihood of poor nutrition literacy. Only 10% of the subjects who participated recognized that lack of education regarding diabetes treatment was a barrier to diabetes care.

Our results also showed that the subjects who scored in the likelihood of poor nutrition literacy category had an average higher HbA1c than those who scored in the possibility of poor nutrition literacy. These results were statistically significant, with a mean difference in HbA1c of 1.9 % ( CI- 0.6-3.0, p=0.005). Poor nutrition literacy could be a barrier to diabetes control. Assessment of nutrition literacy before the start of medical nutrition therapy and after could be helpful in understanding and guiding patients to modify their diet and achieve treatment goals optimally.

Furthermore, this difference in HbA1c, which we would expect to be even more if comparing patients in the likelihood of poor nutrition literacy to those in the range of good nutrition literacy, could potentially have a significant clinical impact on health outcomes and healthcare expenditure. It is essential to highlight that currently available medications for the treatment of diabetes decrease HbA1c approximately by 0.5-1.5%, and based on our results, medical nutrition therapy could be of a higher impact than medications [24]. 

The NLit assessment tested six nutrition domains. Subjects scored the worst in the Food numeracy and Labels. Data regarding health literacy, food label interpretation, and food numeracy is controversial. Some studies have shown that patients with limited health literacy have greater difficulty interpreting food labels and are less likely to refer to food labels [25-27]. However, other studies have shown a negative association between health literacy and food labels [28-30]. Nevertheless, focused didactics on food numeracy and label instruction may be a high yield topic during medical nutrition therapy based on our results.

There are several limitations to this study. Some diabetes-related data was self-reported due to a lack of medical records access. Monthly resources and advertising limited our class size. None of our subjects scored in the likelihood of good nutrition literacy category, and we could not assess if there were a significant difference in HbA1c in this subgroup. Longitudinal studies and post-intervention analyses were not possible due to a lack of consistent monthly follow-up. Future studies with a larger sample size may be beneficial to elucidate further understanding of nutrition literacy and how this can improve diabetes outcomes in groups with socioeconomic disparities.

## Conclusions

To our knowledge, this is the first study utilizing the Nutrition Literacy (NLit) assessment to evaluate nutrition literacy in uninsured subjects with diabetes mellitus. This study demonstrated that poor nutrition literacy is associated with worse glycemic control among uninsured patients with diabetes mellitus based on the Nutrition Literacy Assessment. We suggest a possible role in assessing nutrition literacy in patients with diabetes to identify deficiencies that could be improved upon with adequate medical, nutritional therapy. Furthermore, empowering patients with diabetes and providing broader access to nutritional education to improve nutrition literacy could represent a high yield intervention that can improve health outcomes and reduce the cost associated with the disease. More research is needed to understand better how nutritional literacy optimizes or modulates glycemic control among uninsured and insured patients to address health outcomes and disparities.
